# Bacterial hemerythrin domain-containing oxygen and redox sensors: Versatile roles for oxygen and redox signaling

**DOI:** 10.3389/fmolb.2022.967059

**Published:** 2022-08-05

**Authors:** Kenichi Kitanishi

**Affiliations:** Department of Chemistry, Faculty of Science, Tokyo University of Science, Tokyo, Japan

**Keywords:** hemerythrin, non-heme diiron, oxygen senor, redox sensor, signaling, methyl-accepting chemotaxis protein, c-di-GMP

## Abstract

Hemerythrin is an oxygen-binding protein originally found in certain marine invertebrates. Oxygen reversibly binds at its non-heme diiron center, which consists of two oxo-bridged iron atoms bound to a characteristic conserved set of five His residues, one Glu residue, and one Asp residue. It was recently discovered that several bacteria utilize hemerythrin as an oxygen- and redox-sensing domain in responding to changes in cellular oxygen concentration or redox status, and immediately adapt to these environmental changes in order to maintain important physiological processes, including chemotaxis and c-di-GMP synthesis and degradation. This Mini Review focuses on the recent progress made on structural and functional aspects of these emerging bacterial hemerythrin domain-containing oxygen and redox sensors, revealing characteristic features of this family of proteins.

## Introduction

Bacteria sense various environmental changes to survive and respond immediately in order to maintain cellular homeostasis. To this end, bacteria utilize many sensor proteins that have evolved for sensing various specific environmental stimuli. In particular, oxygen and redox sensor proteins often contain a redox-active metal-containing prosthetic group such as a heme, non-heme iron or iron-sulfur cluster for sensing intracellular and extracellular oxygen concentrations and redox status.

In general, sensor proteins are composed of an N-terminal cofactor-containing sensing domain and C-terminal functional domain. Association/dissociation of oxygen molecules with/from the metal center (in the case of an oxygen sensor) or reduction/oxidation of the metal center (in the case of a redox sensor) induces structural changes in the sensing domain. These structural changes are then transmitted to the functional domain, thereby switching on/off transcription or catalytic reactions (Igarashi et al., 2011; Martínková et al., 2013; Shimizu et al., 2015).

**FIGURE 1:**
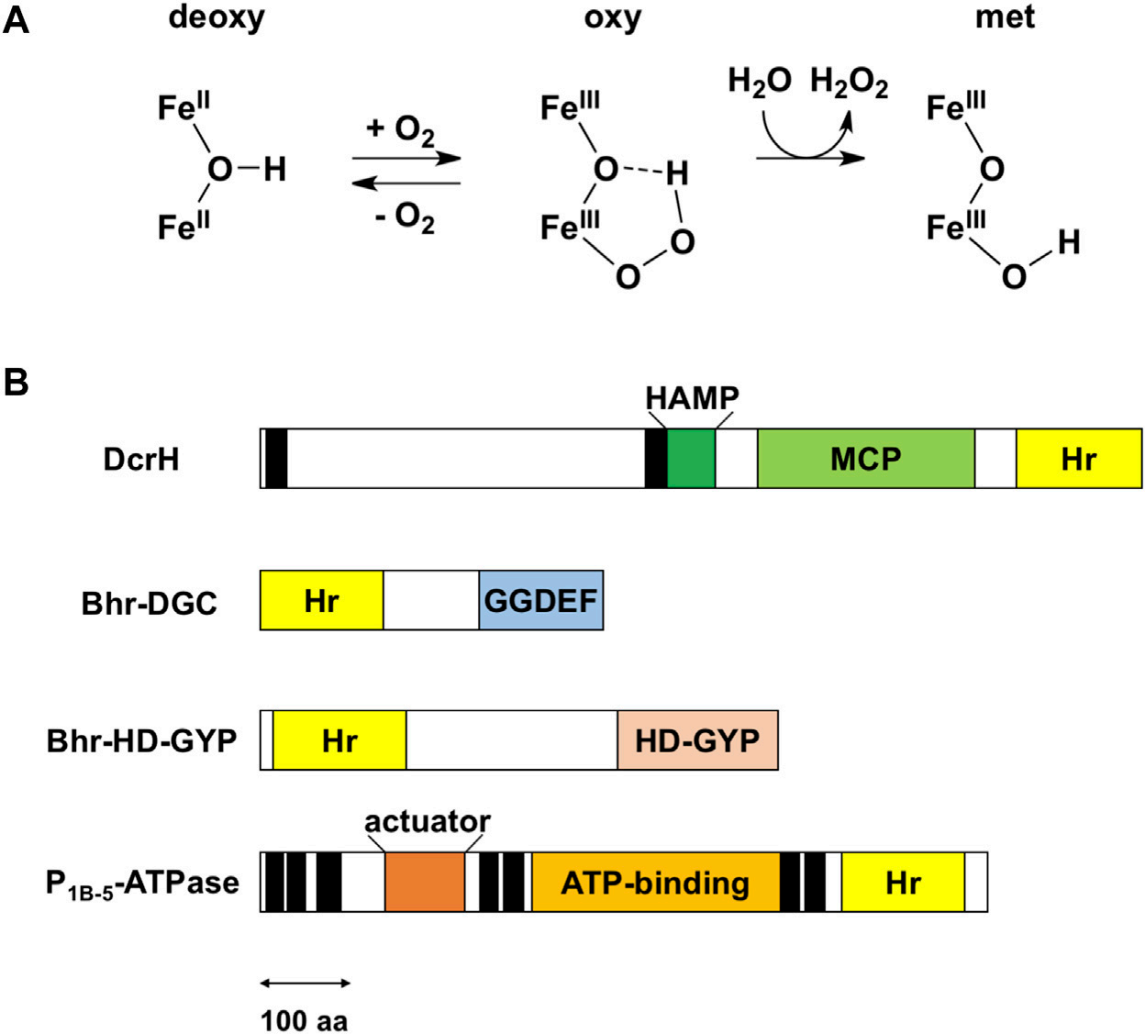
Reaction of the non-heme diiron center and domain structure of bacterial hemerythrin domain-containing sensors. **(A)** Reaction scheme for the non-heme diiron center. Upper and lower irons are Fe1 and Fe2, respectively. The iron ligand residues are omitted for the sake of clarity. The oxy form is a transient intermediate in reactions involving bacterial hemerythrin domain-containing sensors. **(B)** Domain structures of bacterial hemerythrin domain-containing sensors that have been characterized so far, namely *D. vulgaris* DcrH (UniProt ID: Q726F3), *V. cholerae* Bhr-DGC (UniProt ID: Q9KSP0), *Ferrovum* sp. PN-J185 Bhr-HD-GYP (UniProt ID: A0A149VUS3), and *A. cellulolyticus* P_1B-5_-ATPase (UniProt ID: A0LQU2). Transmembrane helices are shown as black rectangles. In DcrH, HAMP, MCP, and hemerythrin domains are located in cytoplasm, whereas the region between two transmembrane helices is located in periplasm. In P_1B-5_-ATPase, actuator, ATP-binding, and hemerythrin-like domains are located in cytoplasm. The double-headed arrow represents a length corresponding to 100 amino acid (aa) residues. Hr, hemerythrin.

Hemerythrin is an oxygen-binding protein originally found in certain marine invertebrates such as *Phascolopsis gouldii* (Stenkamp, 1994). Oxygen reversibly binds the non-heme diiron center of this protein. This center consists of two oxo-bridged iron atoms and is accommodated in a four-helix bundle—and is specifically bound to a characteristic conserved set of five His residues, one Glu residue and one Asp residue with the signature sequence motif H-HxxxE-HxxxH-HxxxxD (Supplementary Figure S1). Three of the His residues bind to one of the irons (Fe1), the other two His residues bind the other iron (Fe2), and the Glu and Asp residues bridge the irons. Oxygen binds end-on to the Fe2 iron as a hydroperoxide (Figure 1A), which is a unique property of hemerythrin.

It was recently discovered that several bacteria utilize hemerythrin as an oxygen- and redox-sensing domain in response to changes in cellular oxygen concentration or redox status, and immediately adapt to these environmental changes in order maintaining cellular homeostasis. Bacterial hemerythrins are categorized into two groups: single-domain hemerythrins and chimeric hemerythrins containing N- or C-terminal functional domains (Bailly et al., 2008; French et al., 2008). The latter group constitutes bacterial hemerythrin domain-containing oxygen and/or redox sensor proteins, which is the subject of this Mini Review. Of these proteins, *Desulfovibrio vulgaris* DcrH, a fusion with methyl-accepting chemotaxis protein (MCP), is the best studied (Xiong et al., 2000; Isaza et al., 2006). In addition to DcrH, several new members of this class of proteins, including P_1B-5_-type ATPase, Bhr-DGC, and Bhr-HD-GYP, have been described in recent years (Traverso et al., 2010; Schaller et al., 2012; Kitanishi et al., 2020). The sensors characterized so far are classified functionally as MCPs, proteins involved in c-di-GMP synthesis and degradation, and P-type ATPases (Figure 1B), as described in detail below.

## Methyl-accepting chemotaxis protein: DcrH

Chemotaxis is the process by which bacteria adapt to their environment, specifically by moving toward or away from specific attractants and repellants, respectively. This process is mediated by MCPs, which are membrane-associated receptors whose recognition of a target molecule in each case triggers a phosphorylation/methylation cascade that ultimately regulates the flagellar motor of the bacterium. DcrH is an MCP containing a hemerythrin domain from the anaerobic sulfate-reducing bacterium *D. vulgaris* (Xiong et al., 2000). DcrH is the first example discovered of hemerythrin in bacteria and DcrH is the first characterized example of this family of bacterial hemerythrin domain-containing oxygen and/or redox sensor proteins (Xiong et al., 2000). DcrH contains, from its N-terminus to C-terminus, a periplasmic membrane domain, HAMP (present in histidine kinases, adenylate cyclases, methyl-accepting proteins, and phosphatases) domain, MCP domain and hemerythrin domain (Figure 1B).

Although spectroscopic and crystallographic studies have revealed the nature of the non-heme diiron center of the isolated hemerythrin domain of DcrH, the mechanism of the signal transduction from the hemerythrin domain to the MCP domain in the full-length protein is not fully understood (Xiong et al., 2000; Isaza et al., 2006). This shortcoming is in part due to most of the works on DcrH having been carried out by using the isolated hemerythrin domain of DcrH rather than the full-length protein, which is difficult to purify due to its insolubility resulting from the presence of the transmembrane domain. Considering the putative cytoplasmic localization of the hemerythrin domain, DcrH has been proposed to play a role in negative aerotaxis (Xiong et al., 2000). Further biochemical assay using purified proteins in a reconstituted system and phenotypic assay using *D. vulgaris* by overexpression and/or knockout of the *dcrH* gene will be required to establish the role of the hemerythrin domain.

In general, determining kinetics parameters for oxygen binding is important for understanding the oxygen-binding properties of a protein. A rate constant of 5.3 × 10^8^ M^−1^s^−1^ for association of oxygen with DcrH (*k*_on_) and a rate constant of 160 s^−1^ for the corresponding oxygen dissociation (*k*_off_) have been measured, with a subsequent dissociation constant (*K*_d_) of 0.3 µM (Onoda et al., 2011)—indicative of a relatively strong binding of oxygen to the non-heme diiron center of DcrH, and for example stronger than that to *P. gouldii* hemerythrin (*k*_on_ = 3.3 × 10^6^ M^−1^s^−1^, *k*_off_ = 51 s^−1^, and *K*_d_ = 15 µM) (Farmer et al., 2000). This high affinity results from the fast association, which is made possible by a hydrophobic tunnel that accelerates the access of the O_2_ ligand to the non-heme diiron site. The autooxidation of DcrH (*t*_1/2_ = 22 min) has been measured to be 54 time faster than that of *P. gouldii* hemerythrin (*t*_1/2_ = 20 h) (Farmer et al., 2000; Onoda et al., 2011)—indicating a relative instability of the oxy form of DcrH toward autooxidation, a feature also due to the large substrate tunnel. These results suggest that DcrH is involved in oxygen sensing.

Surprisingly, a mixed-valence state, semimet_R_, was found to be stable for over 1 week (Onoda et al., 2011). This semimet_R_ form is produced from a one-electron reduction of the met form and is regarded as a transient intermediate. These species have not been observed for bacterial hemerythrin domain-containing sensors other than DcrH. The physiological relevance of this short-lived intermediate is currently unknown.

**FIGURE 2:**
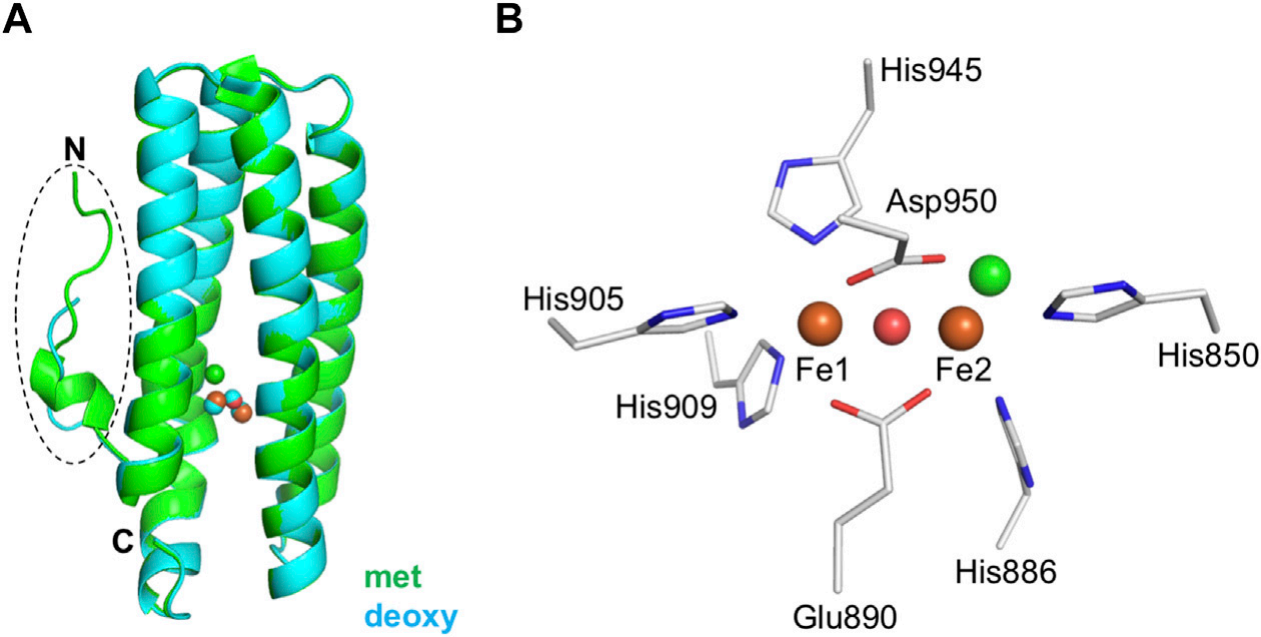
Structural features of the hemerythrin domain of the bacterial hemerythrin domain-containing sensor DcrH. **(A)** Structural comparison of the hemerythrin domain in its met (green, PDB entry 2AWY) and deoxy (cyan, PDB entry 2AWC) forms. The N-terminal loop is circled. **(B)** An enlarged depiction of the non-heme diiron center in its met form (PDB entry 2AWY). Iron atoms and bridging oxo are shown as orange and red spheres, respectively. Chloride (green sphere) is bound to Fe2 in the non-heme diiron center.

Crystal structures of diferric (met), azido-diferric (azidomet), and diferrous (deoxy) complexes of the isolated hemerythrin domain of DcrH were determined to resolutions of 2.1, 1.5, and 2.2 Å, respectively (Isaza et al., 2006). These crystal structures were the first ones determined for any bacterial hemerythrin domain-containing sensor. Later, higher-resolution structures of met, and semimet_R_ forms were determined to resolutions of 1.4 and 1.8 Å, respectively (Onoda et al., 2011). The overall structure of the isolated hemerythrin domain of DcrH is similar to that of invertebrate hemerythrin, exhibiting the classical invertebrate hemerythrin fold consisting of an N-terminal loop attached to an up-down four-helix bundle with the non-heme diiron site buried (Figure 2A). These structures revealed the nature of the non-heme diiron site, in which three His residues bind to one of the irons (Fe1), and two other His residues bind to the other iron (Fe2) (Figure 2B). The Glu and Asp residues bridge the irons, and a chloride ion (Cl^−^) was observed to be bound at the position that oxygen would normally bind.

More detailed comparisons of the met, azidomet, and deoxy DcrH crystal structures showed a redox-dependent conformational change—that is, with the N-terminal loop highly ordered in both the met and azidomet forms, but disordered in the deoxy form—and ligand (azide) binding not inducing conformational change (Isaza et al., 2006). Furthermore, there is no significant difference between the conformations of the met and semimet_R_ structures, suggesting that the structural change in the loop region is related to reduction of the semimet_R_ form to the deoxy form (Onoda et al., 2011). Thus, DcrH has been proposed to transduce a redox-dependent conformational change in the flexible N-terminal loop to the neighboring methylation domain in the full-length receptor (Isaza et al., 2006). However, without structural information for the full-length protein, the mechanism underlying activation of the functional domain in response to oxygen binding to and/or a redox change in the non-heme diiron site of the hemerythrin sensor domain remains unclear.

By engineering the first or second coordination sphere of the non-heme diiron site, attempts to convert the function of DcrH from that of an O_2_-binding protein to that of an O_2_-activating enzyme have also been made (Okamoto et al., 2013, 2014). Indeed, introducing an additional His residue near the non-heme diiron site in DcrH enabled H_2_O_2_-dependent oxidation of external substrates, namely guaiacol and 1,4-cyclohexadiene. These studies may, in future investigations, lead to the generation of more efficient catalysts for external substrates.

## Synthesis and degradation of c-di-GMP

Bis-(3′,5′)-cyclic dimeric guanosine monophosphate (c-di-GMP) is a ubiquitous intracellular second messenger that regulates cell motility, biofilm formation, and other processes in bacteria (Hengge, 2009; Schirmer and Jenal, 2009; Römling et al., 2013). Intracellular levels of c-di-GMP control switching between the motile planktonic and sedentary biofilm-associated lifestyles of bacteria, with low and high intracellular c-di-GMP levels correlating with motile and sessile phenotypes, respectively. The synthesis of c-di-GMP from two molecules of GTP is catalyzed by diguanylate cyclases (DGCs) containing a GGDEF domain, and the degradation of c-di-GMP into 5′-phosphoguanylyl-(3′,5′)-guanosine (pGpG) and/or two molecules of GMP is catalyzed by phosphodiesterases (PDEs) containing either an EAL or HD-GYP domain. These domains are often fused with various sensor domains for detecting a specific stimulus or ligand. With respect to bacterial hemerythrins, Bhr-DGC and Bhr-HD-GYP have been characterized so far, and contain a C-terminal GGDEF domain and HD-GYP domain, respectively, as described in detail below.

### Synthesis: Bhr-DGC

*Vibrio cholerae* Bhr-DGC is the first demonstrated example of an enzymatically active full-length protein containing a bacterial hemerythrin domain and showing redox-dependent catalysis (Schaller et al., 2012). Bhr-DGC contains an N-terminal hemerythrin domain and C-terminal GGDEF domain (Figure 1B). Although the structure of Bhr-DGC has not yet been determined, the protein was shown to be dimeric in solution. DGC activity requires at least formation of a dimer, the minimal functional unit of DGC. Autooxidation from the diferrous form to the diferric form was observed to occur within 1 min, and without detection of the oxy form—both unlike in the case of DcrH and marine invertebrate hemerythrins. The DGC activity (0.77 min^−1^) of the diferrous (Fe^II^Fe^II^) form was measured to be 10 times higher than that of the diferric (Fe^III^Fe^III^) form (0.073 min^−1^), demonstrating a redox-dependent catalysis. The air-reoxidized protein was observed to revert to the lower activity, indicative of the reversibility of this redox interconversion. The azido-diferric form is also inactive. Thus, the catalytic activity is regulated by redox change, but not ligand binding. In addition, overexpression of Bhr-DGC in *Escherichia coli* cells was found to induce biofilm formation. Overall, Bhr-DGC catalyzes the formation of c-di-GMP from GTP to regulate biofilm formation in the low-oxygen environment in *V. cholerae*.

### Degradation: Bhr-HD-GYP

Recently, Bhr-HD-GYP was discovered from the iron-oxidizing Gram-negative bacterium *Ferrovum* sp. PN-J185, as the second example of a full-length protein containing a bacterial hemerythrin domain (Kitanishi et al., 2020). This protein contains an N-terminal hemerythrin domain and C-terminal HD-GYP domain (Figure 1B), and was suggested to be an elongated dimer in solution. Interestingly, Bhr-HD-GYP contains four iron atoms per subunit, indicating that both its hemerythrin and HD-GYP domains contain non-heme diiron sites that function in oxygen/redox sensing and catalysis, respectively. The HD-GYP domain requires two or three equivalents of divalent metals for catalysis (Galperin and Chou, 2022). Non-heme diiron-binding residues at the hemerythrin domain are completely conserved (Supplementary Figure S1). However, unlike marine invertebrate hemerythrins, Bhr-HD-GYP was observed to not form an oxygen adduct, attributed to rapid autoxidation. The reduced ferrous iron form of the protein was found to catalyze the hydrolysis of c-di-GMP to pGpG (6.2 min^−1^), whereas no activity was detected for the oxidized ferric iron form. The azide-bound form was also observed to be inactive. This trend of redox-regulated, but not ligand-regulated, catalytic activity is similar to that of Bhr-DGC. This redox interconversion was observed to be reversible, suggesting that Bhr-HD-GYP is important for redox sensing *in vivo*. The currently available data suggest that Bhr-HD-GYP regulates the switch between the motile planktonic and sedentary biofilm-associated lifestyles in response to changes in cellular redox status or oxygen concentration.

Note that Bhr-HD-GYP is currently the only known bacterial hemerythrin domain-containing sensor phosphodiesterase. No other type of phosphodiesterase containing an EAL domain has yet been discovered.

## P-type ATPase

P_1B_-type ATPase is a member of a family of integral membrane proteins that use the energy of ATP hydrolysis to transport transition metal ions across cell membranes, and is named after the formation of a phosphorylated intermediate during catalysis. Some P_1B-5_-ATPases (proteins constituting the fifth member of P_1B_-ATPase) contain each a C-terminal hemerythrin-like domain (Figure 1B). Biophysical and biochemical properties of P_1B-5_-ATPases first from the Gram-positive cellulolytic thermophile *Acidothermus cellulolyticus* (Traverso et al., 2010) and later from the root nodule bacterium *Sinorhizobium meliloti* (Zielazinski et al., 2013) have been characterized so far. P_1B-5_-ATPases have each at least six transmembrane helices, an actuator domain, an ATP-binding domain and a hemerythrin-like domain (Figure 1B). The last three transmembrane helices have been proposed to form a binding site for metal substrate. Interestingly, the motif of iron ligand residues of the non-heme diiron center of these hemerythrin-like domains appears to be H-HxxxE-HxE-HxxxxD instead of the canonical iron ligand motif H-HxxxE-HxxxH-HxxxxD that is conserved in marine invertebrate hemerythrins and other bacterial hemerythrin domain-containing sensors described above (Supplementary Figure S1). Autooxidation of *A. cellulolyticus* P_1B-5_-ATPase to the met form is very rapid, as reported for other bacterial hemerythrin domain-containing sensors, whereas the oxy form of *S. meliloti* P_1B-5_-ATPase is unusually stable, with a long half-life for autooxidation. This difference between the bacterial species may be due to their different habitats. However, it is currently unclear whether the role of the hemerythrin domain of P_1B-5_-ATPase is oxygen/redox sensing or iron sensing. Considering the putative oxygen-binding function of P_1B-5_-ATPase and its unique non-heme diiron site, it remains unclear whether this protein should be categorized as a bacterial hemerythrin domain-containing sensor.

## Characteristic features of bacterial hemerythrin domain-containing sensors

What distinguishes the family of bacterial hemerythrin domain-containing sensors? Despite bacterial hemerythrin domain-containing sensors and invertebrate hemerythrins being very similar at the sequence and structure levels, summarizing the family of bacterial hemerythrin domain-containing sensors revealed some distinctive features: 1) conserved iron ligand residues, 2) fusion with an N- or C-terminal functional domain in a single polypeptide, 3) larger substrate tunnel, 4) relatively rapid autooxidation, and 5) oxygen-binding and/or redox-dependent catalysis (Supplementary Table S1).

Bacterial hemerythrin domain-containing sensors exhibit considerable similarities to marine invertebrate hemerythrins, but there are also significant differences. Except for DcrH, the oxy state in this type of sensor protein is generally unstable, unlike the case for invertebrate hemerythrins, as no oxygen adduct has been observed for Bhr-DGC and Bhr-HD-GYP. Autoxidation of these bacterial hemerythrin sensors, including DcrH, Bhr-DGC, and Bhr-HD-GYP, occurs much more rapidly than does that of invertebrate hemerythrins—and this feature at least in the case of DcrH is apparently due to its large observed substrate tunnel, which facilitates rapid oxygen binding and solvent access to the oxygen-binding pocket (Isaza et al., 2006). This tunnel is oriented perpendicular to the long axis of the four-helix bundle and is linked to the binding site at Fe2 of the non-heme diiron center, providing a reasonable rationale for rapid oxygen binding and autooxidation (Isaza et al., 2006).

This rapid autooxidation might suggest that bacterial hemerythrin is more of a redox sensor than an oxygen sensor. However, rapid autooxidation followed by oxygen binding would lead one to think that bacterial hemerythrin sensors could function as both redox and oxygen sensors. This combination of redox- and oxygen-regulated catalytic activity is similar to that observed for several heme-based oxygen sensor proteins in that binding of oxygen to the Fe^II^ heme in the sensor domain regulates its catalytic activity (Martínková et al., 2013; Shimizu et al., 2015). Because oxygen can only bind to the Fe^II^ state of heme, and not to Fe^III^, these proteins are likely both oxygen and redox sensors. Therefore, bacterial hemerythrin domain-containing sensors also function as both oxygen and redox sensors upon binding of oxygen to the non-heme diiron site in the hemerythrin domain and possible subsequent autooxidation. Overall, oxygen binding and redox change in the non-heme diiron center in the hemerythrin domain regulate the catalytic activity in the functional domain. In addition, iron ligands residues of non-heme diiron site are all completely conserved except for P_1B-5_-ATPase. Some residues for forming a hydrophobic substrate tunnel are also conserved. However, the role of these residues has not yet been examined using site-directed mutagenesis.

## Concluding remarks

Since the discovery of DcrH, the first member of the family of bacterial hemerythrin domain-containing sensors to be discovered, this family has been expanding and several new members have been discovered in recent years. This Mini Review has summarized recent progress on the structural and functional aspects of bacterial hemerythrin domain-containing sensors, revealing some common characteristic features of members of this family. However, to fully understand their mechanism of action at the atomic level, visualizing the structural difference(s) between active and inactive states of the full-length protein is required, and is the next challenge to be addressed.

In addition to bacterial hemerythrin domain-containing sensors, single-domain hemerythrins from bacteria have been identified and characterized recently, for example, microoxic hemerythrin from *Pseudomonas aeruginosa* (Clay et al., 2020). Furthermore, hemerythrin-like proteins with other activities such as catalase activity (Ma et al., 2018) have also been identified and characterized recently.

Finally, it should be noted that a search of the genomic database shows a bacterial hemerythrin domain in a wide range of bacteria (Bailly et al., 2008; French et al., 2008), where it appears to function as a universal oxygen- and/or redox-sensing domain module. We expect new members of this family to be discovered in the near future, which would expand our understanding of this family. Further structural and functional characterizations may lead to an understanding of the characteristic features of these increasing numbers of sensors. In addition, engineering non-heme diiron centers is expected to lead to the generation of new types of biocatalysts and FRET sensors for monitoring cellular oxygen levels and redox state.
