# Supplementary material for: Bacterial hemerythrin domain-containing oxygen and redox sensors: Versatile roles for oxygen and redox signaling
Source: Front Mol Biosci. 2022 Aug 5;9:967059. doi: 10.3389/fmolb.2022.967059 (PMC9388753; doi:10.3389/fmolb.2022.967059)
Supplement: Supplementary file 2 [file Image1.PDF]

Supplementary Material

Supplementary Table 1. Characteristic features of bacterial hemerythrin domain-containing sensors. The canonical non-heme diiron site is H-HxxxE-HxxxH-HxxxxD, whereas the noncanonical motif is H-HxxxE-HxE-HxxxxD in *A. cellulolyticus* P_{1B-5}-ATPase.

	Source	Location of Hr domain	Non-heme diiron site	Function	Active form	Inactive form
DcrH	*D. vulgaris*	C	canonical	MCP	not reported	
Bhr-DGC	*V. cholerae*	N	canonical	DGC	deoxy	met, azidomet
Bhr-HD-GYP	*Ferrovum* sp. PN-J185	N	canonical	PDE	deoxy	met, azidomet
P_{1B-5}-ATPase	*A. cellulolyticus*	C	noncanonical	ATPase	not reported	

```
                                           850
DcrH           826 DTGDADVLVKWSEDLAN-LPSIDTQHKRLVDYINDLYRAARRR-----DMDKAREVFDAL 879
Bhr-DGC          1 -----MQSFKWDQYFETGLEEVDEQHQSLVNIVNRYSSLLAENH---VSLDEIRLALFEL  52
Bhr-HD-GYP      14 NTKKSVDIFPWNEYFKIGIEEIDKQHEKLVGILNEVATHVSFN----SKLPELQDIIEKL  69
P1B-5-ATPase   619 LLPGTRHTVGLATADQAVAERLRAEHDVVRSVGQRLRVVADALAPCREDFSALEDLVGEL 678
Hr               1 MGFPIPDPYVWDPSFRTFYSIIDDEHKTLFNGIFHLA------------IDDNADNLGEL  48
                                        :  :*. : .                  :      .  *

                        886 890                  905 909
DcrH           880 KNYAVEHFGYEER----LFADYAYP--EATRHKEIHRRFVETVLKWEKQLAAGDPE---- 929
Bhr-DGC         53 SRYSEYHFKEEEK----LMREVGISALHLEEHIQVHRTFMSEVFSMQAFIHDVDDR---- 104
Bhr-HD-GYP      70 VDYTQYHFKTEES----LWEKYLKNDSSAILHKKSHDRFIEKINAIKLHADNTPTEN--- 122
P1B-5-ATPase   679 EAVLLPHERAEEDQLLPIVARALRSSDVVAGLSRAHAEIEHYVRRLRRLLTMVGGEPESD 738
Hr              49 RRCTGKHFLNEQV----LMQASQYQ--FYDEHKKEHETFIHALDNWKG------------  90
                         *   *:     :               . *  :   :   .

                                    945  950
DcrH           930 ---VVMTTLRGLVDWLVNHIMKED--KKYEAYLRERGVS 963
Bhr-DGC        105 ---SAVQLLEFLIHWLAYHILGIDQNMARQVIAIRSGMS 140
Bhr-HD-GYP     123 ---IINDLLGYLTNWLVEHILEHDRELSYIVYGIQCGLT 158
P1B-5-ATPase   739 DVIEARRLLYGLHAVLDLHNAEEDEIAFALLPDAGQRNT 777
Hr              91 -------DVKWAKSWLVNHIKTIDFKYKGKI-------- 114
                           :      *  *    *
```

Supplementary Figure 2. Shown is the amino acid sequence alignment of the hemerythrin domain of bacterial hemerythrin domain-containing oxygen/redox sensor proteins, *D. vulgaris* DcrH (residues 826-963) (UniProt ID: Q726F3), *V. cholerae* Bhr-DGC (residues 1-140) (UniProt ID: Q9KSP0), *Ferrovum* sp. PN-J185 Bhr-HD-GYP (residues 14-158) (UniProt ID: A0A149VUS3), and *A. cellulolyticus* P_{1B-5}-ATPase (residues 619-777) (UniProt ID: A0LQU2), and *P. gouldii* hemerythrin (Hr) (UniProt ID: P02244). Iron ligand residues are shown in red.
